# Pros and Cons of Dietary Vitamin A and Its Precursors in Poultry Health and Production: A Comprehensive Review

**DOI:** 10.3390/antiox12051131

**Published:** 2023-05-20

**Authors:** Rifat Ullah Khan, Aamir Khan, Shabana Naz, Qudrat Ullah, Nikola Puvača, Vito Laudadio, Domenico Mazzei, Alireza Seidavi, Tugay Ayasan, Vincenzo Tufarelli

**Affiliations:** 1 Faculty of Animal Husbandry and Veterinary Sciences, College of Veterinary Sciences, The University of Agriculture, Peshawar 25000, Pakistan; rukhan@aup.edu.pk; 2 Directorate General (Research), Livestock and Dairy Development Department, Khyber Pakhtunkhwa, Peshawar 59000, Pakistan; aamirkhanawan1@gmail.com; 3 Department of Zoology, Government College University, Faisalabad 38000, Pakistan; drshabananaz@gcuf.edu.pk; 4 Faculty of Veterinary and Animal Sciences, The University of Agriculture, Dera Ismail Khan 29220, Pakistan; qudratmahsud@gmail.com; 5 Faculty of Economics and Engineering Management, University Business Academy in Novi, 21000 Novi Sad, Serbia; nikola.puvaca@fimek.edu.rs; 6 Department of Precision and Regenerative Medicine and Jonian Area, Section of Veterinary Science and Animal Production, University of Bari Aldo Moro, 70010 Valenzano, Italy; vito.laudadio@uniba.it (V.L.); domenico.mazzei@uniba.it (D.M.); 7 Department of Animal Science, Rasht Branch, Islamic Azad University, Rasht 41335-3516, Iran; alirezaseidavi@iaurasht.ac.ir; 8 Department of Organic Farming Business Management, Kadirli Faculty of Applied Sciences, University of Korkut Ata, Osmaniye 80000, Turkey; tayasan@gmail.com

**Keywords:** vitamin A, health, growth, poultry, antioxidant

## Abstract

Vitamin A is a fat-soluble vitamin that cannot be synthesized in the body and must be obtained through diet. Despite being one of the earliest vitamins identified, a complete range of biological actions is still unknown. Carotenoids are a category of roughly 600 chemicals that are structurally related to vitamin A. Vitamin A can be present in the body in the form of retinol, retinal, and retinoic acid. Vitamins are required in minute amounts, yet they are critical for health, maintenance, and performing key biological functions in the body, such as growth, embryo development, epithelial cell differentiation, and immune function. Vitamin A deficiency induces a variety of problems, including lack of appetite, decreased development and immunity, and susceptibility to many diseases. Dietary preformed vitamin A, provitamin A, and several classes of carotenoids can be used to meet vitamin A requirements. The aim of this review is to compile the available scientific literature regarding the sources and important functions, such as growth, immunity, antioxidant, and other biological activities of vitamin A in poultry.

## 1. Introduction

Vitamin A is an alicyclic ring-containing group of unsaturated monohydric alcohols. Vitamin A is water-insoluble but fat-soluble [1]. Retinol, retinal, and retinoic acid are all forms of vitamin A. Vitamin A’s principal biological activities include the maintenance of eyesight, development, and epithelial and mucous tissue integrity [2]. It is vital to remember that vitamin A and its metabolites are lipids, which means that they are insoluble in the body’s aqueous environment. As a result, vitamin A is discovered attached to one of a variety of diverse but specialized, vitamin A-binding proteins within cells and in the extracellular space or is present within intracellular lipid droplets. Stepp et al. [3] reported the presence of a chemical that is required for life, which is abundant in egg yolk. This molecule was first identified as part of the “fat-soluble” fraction “A” and later dubbed vitamin A [4,5].

## 2. Precursors of Vitamin A

The ability of natural carotenoids to form retinol (Vitamin A) through the action of the dioxygenase enzyme is known as provitamin A activity [6]. The α- and β-carotene are the fundamental precursors of vitamin A. Any pigment with at least one intact ionone ring in its structure can be classified as a provitamin A carotenoid [7]. Carotenoids can be found in a variety of fruits and vegetables, including carrots, pumpkins, apricots, sweet potatoes, and beans. Carotenoids are classified primarily based on their chemical structure or functional properties. Carotenoids are divided into primary and secondary carotenoids based on their chemical structure. Carotenoids that exist as pure hydrocarbons are referred to as carotenes. Furthermore, xanthophylls are carotenoids with oxygen as a functional group in their structure. Predominant carotenoids are lycopene, β-cryptoxanxanthin, zeaxanthin, lutein, and canthaxanthin. Carotenoids are colorful pigments that produce multiple colors and are mostly found in plants. Because of their positive impact on health, there has recently been extensive interest in the research on using carotenoids as a feed supplement in the poultry sector. Carotenoids are powerful antioxidants that can reduce the negative effects of oxidative stress through a variety of mechanisms, including free radical scavenging and downregulation of signaling pathways. Feeding trials in poultry have shown that it improves productivity and reproduction, as well as the oxidative stability of poultry products such as eggs and meat [8].

Vitamin A and carotenoids have been widely recognized as physiologically essential elements for poultry health and production during the last two decades. There are only a few types of vitamin A, and their metabolism is tightly controlled by a network of enzymes, binding proteins, and storage vectors, most likely due to its toxicity at greater doses [9].

Retinoid is referred to both natural and synthetic forms of retinol [10]. Vitamin A is present in plants in the form of β-carotene, which is a precursor of vitamin A. β-carotene, a naturally occurring compound with vitamin A-like activity, is a member of the carotenoid family. During intestinal digestion, carotenoids with vitamin A biological activity are transformed into retinol [11]. For all vertebrates, including chickens, vitamin A is a critical nutrient. This vitamin is important for eyesight, epithelium surface maintenance, immunological competence, growth, development, and reproduction, among other activities [2,12,13]. Vitamin A is found in two forms in the diet, retinoids and provitamin A carotenoids, and its availability in the body is determined by a variety of factors, including dietary intake, intestinal absorption, and conversion of pro-VA carotenoids to retinoids. Carotenoids, responsible for the broiler’s distinct skin and egg yolk colors, are critical for consumer acceptance in many countries [14,15,16].

Carotenoids cannot be synthesized by animals; hence, they must be obtained by dietary supplementation with plant products. Dietary carotenoids are cleaved in animals to supply precursors for vitamin A production (a deficit results in blindness) and are important for a variety of physiological activities, such as immunocompetence, antioxidant activity, yolk nutrition of embryos, and UV protection [17,18]. On an industrial scale, the nutritional industry synthesizes five primary carotenoids (e.g., carotene, lycopene, canthaxanthin, zeaxanthin, and astaxanthin) for use in food items, vitamin supplements, and health goods, as well as feed additives for poultry, cattle, fish, and crustaceans [19,20]. The main natural sources of carotenoids used in poultry diets are corn gluten meal, yellow corn, algae meal, and dehydrated alfalfa meal [21]. Carotenoids are provided by photosynthetic and non-photosynthetic organisms.

**Figure 1:**
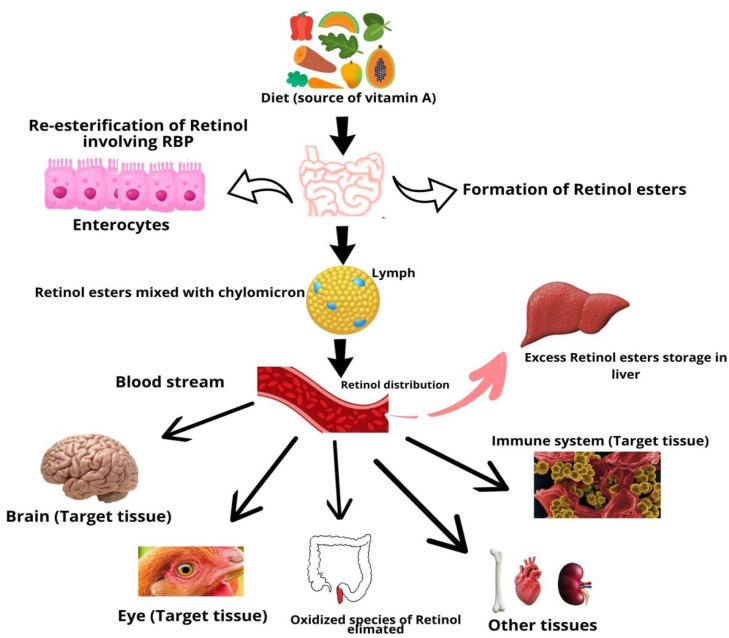
Absorption and metabolism of vitamin A.

Vitamin A is a fat-soluble vitamin that cannot be produced and must be received from the food. The majority of dietary provitamin A carotenoids are hydrolyzed into retinal. In brush border cells, retinal is converted into retinol and packed into chylomicron particles. Lipase enzyme activity in extrahepatic organs partially degrades chylomicrons, resulting in chylomicron remnants, which are subsequently obtained by liver cells and enzymatically cleaved in endosomes to generate free vitamin A [22]. Vitamin A that has been degraded can be re-esterified and transported to stellate cells for storage or excretion (Figure 1).

## 3. Requirements of Vitamin A in Poultry

The NRC recommendations in force were established decades ago, in 1994, and did not take into account genetic selection, which has significantly improved growth and performance since then [23]. Many other factors are also responsible for increasing the requirement, such as diet composition, environmental temperature, infectious diseases, biological variations, stress, parasites, and bioavailability. According to NRC [24], the vitamin A requirement for broilers, growing layers, and developing geese is 1500 IU per kg of feed, but the need for breeding and laying hens ingesting 100 g per day is 3000 IU per kg of diet. The requirement for all classes of turkey is 5000 IU per kg. Similarly, the requirement for vitamin A in the growth phases of ducks is 2500 IU/kg, whereas the requirement for breeders is 4000 IU/kg of diets. The beginning and growth requirements for Japanese quail are 1650 IU/kg of food and 3300 IU/kg for breeders. According to Olson [25], most animals require 100 to 200 IU of vitamin A per kilogram of body weight each day. In poultry, the well-known carotenoids having vitamin A-like activities are α-carotene, β-carotene, and cryptoxanthin. According to Tanumihardjo and Howe [26], the provitamin A value of α-carotene and cryptoxanthin is around half that of β-carotene. When compared to other animals, poultry are the most effective converters of β-carotene to vitamin A. Stressful situations, such as excessively hot temperatures, viral infections, and other illness states, can all lead to a decrease in carotene to vitamin A conversion. Coccidiosis in chicken not only destroys vitamin A in the gut but also injures the intestinal wall, resulting in impaired absorption of vitamin A and anorexia for many days [27]. According to McDowell and Ward [23], vitamin A insufficiency is common in extensively parasitized animals that purportedly receive an appropriate quantity of the vitamin. Vitamin A reserves in humans were favorably related to numerous indicators of innate immune activation throughout a wide range of storage, demonstrating that vitamin A increases protection against a variety of illnesses even at concentrations greater than that necessary to maintain normal vision [28]. Wang et al. [29] reported that vitamin A level in offspring has a greater importance than maternal provision. Nonetheless, both should be considered to achieve better meat quality and immunity.

Stress impairs the immune system, increases nutritional requirements, particularly for vitamin A. Friedman and Sklan [30] found that vitamin A intakes three to ten times higher than NRC recommended values resulted in optimum immune responses in growing chicks and turkeys. Large quantities of vitamin A supplementation to layers improved laying performance and immune function during heat stress [31]. The NRC [24] requirement of vitamin A in poultry is summarized in Table 1.

## 4. Feed Intake and Efficiency

It has been suggested that vitamin A has positive effects on nutrient digestibility and plays an essential role in maintaining intestinal health [32]. Recently, Stanquevis et al. [33] reported that vitamin A has positive effects on feed intake and feed efficiency in Japanese quails. According to Chen et al. [34], vitamin A dietary supplementation at 21,600 IU/kg enhanced feed efficiency by raising the egg-to-feed ratio compared to 5400 IU/kg. Mendonca et al. [35] found that the provision of vitamin A at various rates (5000, 10,000, 15,000, 20,000, and 25,000 IU/kg) in the form of retinyl acetate to Hyline white layers for 15 weeks had no negative impacts on feed intake. Lin et al. [31] showed that raising vitamin A levels from 3000 to 9000 IU/kg for a period of 35 days enhanced feed intake in heat-stressed laying hens. According to Safarizadeh and Zaker [36], vitamin A supplementation had no influence on broiler chick feed intake or feed conversion ratio. Abd El-Hack et al. [37] demonstrated that feeding carotenoid to layers at a rate of 16,000 IU/kg of feed enhances feed intake and feed conversion ratio. Savaris et al. [38] showed that feeding carotenoid to broilers at 0 or 3000 IU/kg of feed has a detrimental effect on feed intake. Kaya et al. [39] demonstrated that feeding carotenoid to layers at a rate of 10,000 IU/kg of feed boosted the feed conversion ratio. According to Feng et al. [40], feeding carotenoid to growing ducks at levels ranging from 0 to 2500 IU/kg of feed boosted feed efficiency while above 2500 IU/kg lowered it. The addition of vitamin A benefits the intestinal environment and accelerates nutrient absorption, thereby promoting growth rate [41]. This vitamin also regulates physiological processes in birds, such as metabolism and hormone development [42]. Detailed information on feed intake and efficiency is presented in Table 2.

## 5. Growth Performance in Poultry

Vitamins, as necessary micronutrients, play critical roles in broiler production, impacting egg hatching and chick survival and subsequently influencing broiler growth and development throughout their lives [61]. It has been speculated that Vitamin A supplementation in the diet for a period of 8 weeks improved growth performance, but maternal vitamin A levels affect the initial body weight of offspring after they hatch [29]. A study also reported that maternal deficiency of vitamin A affected the growth and development of gosling [62]. Khoramabadi et al. [32] discovered that greater dosages of vitamin A resulted in superior weight growth and performance levels in broilers. This might be due to vitamin A effect on enhanced nutritional digestibility and its function in preserving intestinal health or integrity. However, high amounts of vitamin A may interfere with vitamin D3 absorption and alter bone formation, resulting in a loss in weight and performance [63]. Liang et al. [50] concluded that vitamin A supplementation had a quadratic–linear relationship with the growth of gosling with a minimum dose of 9000 IU/kg.

Abd El-Hack et al. [37] found that providing 16,000 IU/kg of feed as a carotenoid supplement to the layers enhances body weight and growth performance. Feng et al. [40] demonstrated that dietary supplementation of carotenoid to growing ducks for a period of 21 days at levels ranging from 0 to 2500 IU/kg of feed boosted body weight gain and growth performance, but levels over 2500 IU/kg lowered body weight gain and growth performance. Birds fed a carotenoid-enriched diet improved their performance [64,65]. Wang et al. [29] demonstrated that feeding carotenoids to broilers at a rate of 21,600 IU/kg of feed boosted body weight and growth performance. Similarly, Liang et al. [50] reported that dietary supplementation of 9000 IU/kg provitamin A improved body weight and growth performance in goslings. Chen et al. [34] found that supplementing vitamin A for a period of 9 weeks lowered final body weight and raised average egg mass and average egg weight with 21,600 IU/kg dietary VA and was considerably greater than that of birds fed 5400 IU/kg Vitamin A. According to Safarizadeh and Zaker [36], vitamin A supplementation had no meaningful influence on the body weight and performance of broiler chicks. Vitamin A supplementation (15,000 IU) for a period of 9 weeks resulted in increased live weight growth and performance in broiler chickens [44].

Birds fed a carotenoid-supplemented diet improved their performance and product quality [64,65]. Carotenoids and other plant-derived substances have shown great therapeutic and health-promoting potential in improving poultry bird production performance [66]. Another study found that supplementing canthaxanthin (6 mg/kg) boosted performance and decreased embryonic mortality in broiler breeders [60]. It was discovered that 40 mg/kg dietary lycopene greatly enhanced broiler chick body weight [51]. Recently, Savaris et al. [38] reported that vitamin A supplementation in broilers positively influenced wooden breasts, white striping, and meat color. On the other hand, Xiao et al. [67] reported that vitamin A deficiency had adverse effects on growth and slaughter performance and meat quality. Detailed information on the effects of vitamin A dietary supplementation on growth performance and health is presented in Table 2 and Table 3. Discrepancies among the studies might be due to the presence of variable crude fat content in the feed, as fats may influence the absorption of other nutrients, such as fat-soluble vitamins [68]. Similarly, it has been revealed that discrepancies among the studies could be due to dietary supplementation of vitamin A above the minimum required values, which will impact the nutritional value of poultry products, poultry health, and welfare [69].

## 6. Egg Production and Quality

**Figure 2:**
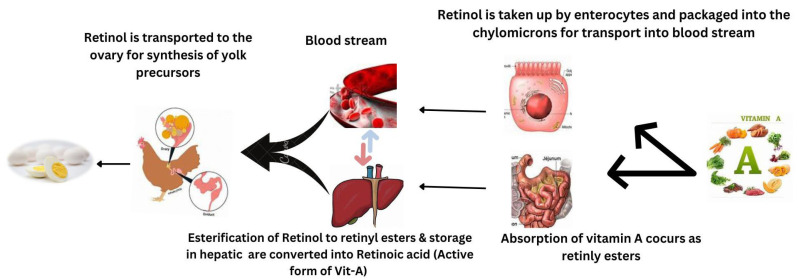
Mechanism of incorporation of retinol in egg yolk.

According to Chen et al. [34], vitamin A supplementation enhanced the laying rate, average egg mass, and average egg weight in broilers fed 21,600 IU/kg compared to birds fed 5400 IU/kg of dietary vitamin A. Mendonca et al. [35] found that providing vitamin A doses ranging from 5000 to 25,000 IU/kg in the form of retinyl acetate to Hy-Line white layers for 15 weeks had no negative impact on egg weight or egg production. However, when vitamin A was added to the basal diet, there was a progressive rise in the incorporation of retinol into egg yolk; the percentage of increase reached 50.6% with 25,000 IU of dietary retinyl acetate/kg. The mechanism of incorporation of retinol in egg yolk is reported in Figure 2.

Significant declines in egg yolk tocopherol concentrations, on the other hand, indicated that vitamin A supplementation had a negative effect. The increase in egg production could be attributed to the fact that giving Vitamin A to birds stimulates ovary and oviduct development and advances the onset of the first oviposition [84]. Second, vitamin A has been linked to estrogen-induced cell proliferation [85]. Third, either alone or in combination with LH or FSH, dietary vitamin A intake stimulates IGF-IR expression to mediate IGF-I and IGF-I activity, promoting follicular growth and development [86,87].

March et al. [57] found a drop in egg weight and a significant decrease in egg production when increased amounts of vitamin A were supplied, ranging from 210,000 to 410,000 IU/kg of feed. Squires and Naber [59] discovered no significant variations in albumen quality between un-supplemented and supplemented (9000 IU/kg) diets. Coskun et al. [58], on the other hand, found that adding 24,000 IU/kg of vitamin A to the diet failed to improve egg production in laying hens after 72 weeks. Lin et al. [31] discovered that raising vitamin A levels from 3000 to 9000 IU/kg for a period of 35 days increased the laying performance and egg weight of heat-stressed laying chickens. The dietary addition of carotenoids to poultry birds may boost production performance while also improving egg and meat quality [64]. Pigments added to poultry feed increase skin, meat, and egg yolk color [88,89]. Umar Faruk et al. [43] reported that nutritional supplementation with canthaxanthin 0–8 mg/kg of feed for a period of 3 weeks increased egg production and quality parameters. Further study showed that feeding laying hens genetically modified corn with increased amounts of cryptoxanthin may increase provitamin A and egg yolk color [90]. In contrast, adding carrot leaves to the chicken diet improves egg yolk color and lutein content [91]. Lycopene pigment (12 mg/kg) supplementation, in combination with some other supplements, improved the production performance [92]. Similarly, supplementation with carotenoids at levels of (2.5, 5.0, and 7.5% tomato pomace) together with other feed additives, effectively reduced lipid oxidation and improved egg yolk color in laying hens [93]. Overdose of vitamin A may produce negative effects on egg production due to its indirect action on thyroid activity [57]. Yuan et al. [56] found that supplementing laying hens with 10,000 to 15,000 IU/kg of vitamin A reduced egg production, egg weight, and egg size. According to Abd El-Hack et al. [34], vitamin A supplementation improved egg production in layers by 16,000 to 15,000 IU/kg dietary supplementation. The discrepancies in the result of egg production could be due to greater amounts of vitamin A, which could have some negative effects on egg production due to its indirect action on thyroid activity [35].

## 7. Immunity

Carotenoids are used by birds and other oviparous animals as a powerful immunomodulatory agent [94]. Carotenoids have been shown to have extraordinary impacts on both acquired and innate immunity in avian species. According to Safarizadeh and Zakeri [36], supplementing with vitamin A enhances the humoral immune system in broiler chickens. Recently, Zhang et al. [95] reported that vitamin A supplementation improved immune function against infectious disease, inhibited viral infection, and suppressed excessive inflammation process. Moreover, Guo et al. [96] concluded that vitamin A supplementation in the diet improved the immune response in necrotic enteritis--challenged birds. It was reported that vitamin A increased antibody responses to T-cell-dependent antigens [97]. Coskun et al. [58] found that vitamin A supplementation had no influence on maternal-derived antibody titer or the histological structure of lymphoid organs. It was found vitamin A supplementation had influence on Newcastle disease antibody titer in laying hens [54]. Lin et al. [31] discovered that raising vitamin A levels from 3000 to 9000 IU/kg for a period of 35 days enhanced the immunological state of heat-stressed layers as well. Sijtsma et al. [98] discovered that vitamin A deficiency diets exacerbated disease severity following experimental NDV infection. Davis and Sell [99] discovered that vitamin A deficiency harmed lymphoid tissue growth and development, and vitamin A-deficient broilers had reduced relative bursa and thymus weights. Vitamin supplementation is essential to antibody production during heat stress, according to Ferket and Qureshi [100]. Vitamin A deficiency has been demonstrated to suppress T-cell proliferative responses [80,81].

Dietary addition of carotenoids to poultry birds may enhance their immunity against various infections [64]. Carotenoid pigments are reported to boost antibody production against the Newcastle disease virus [101]. Carotenoids and other plant-derived substances have shown great therapeutic and health-promoting potential in improving poultry bird production performance [66,102]. Another study found that feeding broilers a high carotenoid diet improved protective immunity against viral disease [103]. In birds, during a period of 4 weeks, carotenoids at levels of 8 or 38 mg/kg had a tendency to concentrate in tissues related to the immune system [104]. Egg yolk lycopene in laying hens at 23 weeks of age rose with increasing dietary lycopene, while dietary lycopene had no effect on immunological response [105]. Detailed information on immunity is presented in Table 3.

Based on Rajput et al. [21], carotenoids have immunomodulatory properties. Carotenoids have a diverse biological role that leads to therapeutic benefits such as immunomodulatory, antibacterial, anti-inflammatory, anticancer, neuroprotective, and anti-diabetic properties [106,107,108]. Lycopene increases immunological response, cell growth, and gene transcription [109]. Phytochemicals contained in fruits and plants, such as lipophilic carotenoids, have been shown to possess anti-inflammatory and antioxidant properties. The majority of these properties have been shown to prevent or impede inflammatory processes as well as oxidative stress [110]. It has been revealed that a suitable level of vitamin A is essential for the secretion of IgA and mucin by regulating the gene expression of cytokines and epithelial growth factors. So sources of discrepancies among the studies might be increased doses as high concentrations of vitamin A inhibit the secretion of IgA as well as the synthesis and secretion of mucus by the chick tracheal epithelium [111], suggesting that vitamin A deficiency or high dose vitamin A is disadvantageous for health and suppress the immunity by suppressing the gene expression of cytokines [82].

## 8. Antioxidants Effects

In the body, free radicals are continually produced, and specific levels of these components are required for proper physiological activities. When their levels surpass normal, they induce peroxidative damage to the cell membrane and organelles [112]. Damage from free radicals includes reactive oxygen species that induce peroxidation of polyunsaturated fatty acids in the cell membrane bilayer, which produces a chain reaction of lipid peroxidation that damages the cell membrane and promotes additional oxidation of lipid and protein membranes. Vitamins A1 (retinol) and A2 (dehydroretinol), as well as various pro-vitamin A molecules, such as alpha and beta carotenes, have been shown to have antioxidant action. According to Surai [113], carotenoids exhibit antioxidant characteristics that scavenge peroxy radicals and singlet oxygen, thus protecting lipids from hydroxyl and superoxide radical assaults. According to McDowell et al. [114], the antioxidant carotenoid, such as beta-carotene, have substantial antioxidant capabilities. Rajput et al. [21] reported that carotenoids exhibit antioxidant and immunomodulatory properties in broilers. Carotenoids have antioxidant characteristics [115], assist in critical biological functions [116], and stimulate the immune response in chickens [117]. For many years, carotenoids have been added to chicken diets as a pigment to achieve the desired color of egg yolk and broiler skin [116]. Vitamin A may also be able to reduce the oxidative damage caused by heat exposure and immunological stress [29]. During the early life of gosling, Liang et al. [118] reported that maternal supplementation of vitamin A enhanced total antioxidant capacity and enzymes and reduced oxidative stress.

**Figure 3:**
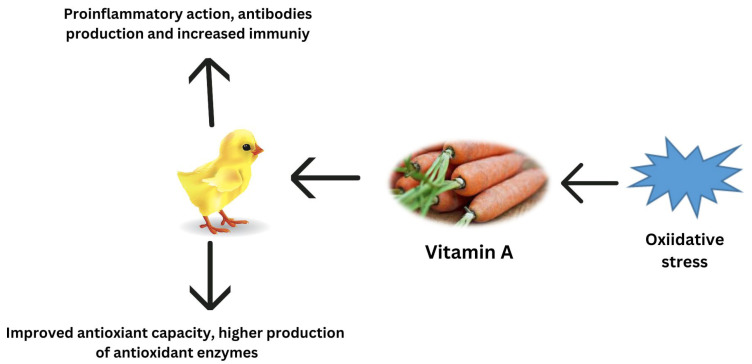
Regulation of antioxidant potential by vitamin A during oxidative stress.

Carotenoids provide several health advantages, including antioxidant and anti-inflammatory properties, as well as playing a significant part in the prevention of certain diseases [119]. Several studies have shown that in pre-hatched and post-hatched birds, carotenoid pigments decrease oxidative stress through various methods, such as activating antioxidant enzymes, quenching free radicals, and blocking signaling pathways [64,120]. According to Yuan et al. [121], the microalgae-derived ketocarotenoid astaxanthin mitigated oxidative stress via the downregulation of inflammatory cytokines, inhibition of the activity of the renin–angiotensin system, antimicrobial effects, and decreasing nuclear factor-kB activation. Among all carotenoids, zeaxanthin, cryptoxanthin, carotene, and lutein are the most powerful pigments for protecting cells from ROS damage [122]. Lycopene’s most significant purpose is to preserve DNA from oxidative stress by scavenging oxygen and decreasing mutations that might cause chronic diseases [123]. According to another study, lycopene decreases heat stress in chickens by increasing the synthesis of phase II cytoprotective enzymes [71]. Furthermore, supplemental lycopene activates the Nrf2/ARE transcription pathway, which reduces oxidative stress and promotes the health of poultry birds [71]. Carotenoids, being highly lipophilic chemicals, are thought to be excellent scavengers of free radicals at the cellular level [124]. The regulation of antioxidant potential by vitamin A during oxidative stress is reported in Figure 3.

Vitamin A supplementation (15,000 IU per kg) for a period of 4 weeks lowered blood malondialdehyde (MDA) concentrations in broiler chickens, which serves as a biomarker of lipid peroxidation [44]. In broiler chicks, 40 mg/kg of dietary lycopene dramatically enhanced total antioxidant capacity (TCA) and decreased hepatic MDA in the liver [51]. Similarly, supplementation with carotenoids at various doses resulted in considerably decreased lipid oxidation in laying hens [93,105,125,126]. According to Liang et al. [50], nutritional supplementation of Goslings with 9000 IU/kg improved antioxidant status via the activity of glutathione peroxidase (GSH-PX), total antioxidant capacity (T-AOC), superoxide dismutase (SOD), and catalase (CAT) and lowered MDA concentration in blood.

Monoghan and Schmitt [127] were the first to characterize the antioxidant potential of vitamin A and carotenoids, reporting that both could preserve lipids from rancidity. Burton and Ingold [128] described the mechanism via which carotenoids may quench lipid radicals in biological membranes for the first time. At least a dozen different types of vitamin A have been extracted, and over 600 different carotenoid compounds have been identified [129]. Retinol may be reversibly converted to retinal in a variety of tissues. Retinal can further be converted to retinoic acid. The retinol-binding protein (RBP) and transthyretin bind to retinol and transport it through the bloodstream. The development of this transport complex is thought to aid in the prevention of the loss of vitamin A–RBP complex through the kidneys.

Although retinol, retinal, and retinoic acid are physiologically active forms of vitamin A, they are all toxic in large quantities, necessitating the storage of excess vitamin A. The predominant storage form of the vitamin is long-chain fatty acid esters, which are produced in the liver by acyl transferase enzymes. Different forms of retinol are stored in specialized storage cells known as stellate cells [130].

Although there are over 600 distinct carotenoids, only about 50 of them exhibit antioxidant action [129]. Retinol’s suppression of peroxidation demonstrated that it is an effective peroxyl radical scavenger [131], with retinol being even more efficient than tocopherol in scavenging peroxyl radicals. The short polyene chain of retinol, compared to tocopherol, allows it to have a better chance of interacting with peroxyl radicals. Using an in vitro peroxidation system, the antioxidant activities of retinoids were rated as retinol > retina > retinyl palmitate > retinoic acid [132]. The discrepancies in the result of antioxidant activity might be due to increased levels of vitamin A in the diet; high concentrations of dietary vitamin A decreased liver concentrations of vitamin E, resulting in increased plasma MDA concentration [113].

## 9. Vitamin A Deficiency and Toxicity

Many studies have found that vitamin A deficiency causes stunted development, decreased resistance to disease, eye lesions, muscle incoordination, decreased egg output, and blood spots in eggs [27,59]. Squires and Naber [59] discovered a high occurrence of blood spots in eggs as a result of a vitamin A-deficient diet. Anorexia, a lack of development, lethargy, weakness, incoordination, emaciation, and ruffled plumage are all symptoms of vitamin A deficiency in chicks. The mucous membranes of poultry’s nasal passage, mouth, esophagus, and pharynx are damaged, resulting in white pustules [27]. Excess vitamin A also impacts the metabolism of other fat-soluble vitamins by competing for transport and absorption. As a result, a significant increase in vitamin A in diet may induce decreased development or egg production by interfering with the absorption of other vitamins than a harmful impact of vitamin A itself. According to Yuan et al. [56], high levels of vitamin A retained in the body are hazardous to chickens. Excess vitamin A causes congenital abnormalities during embryonic development, according to Clagett-Dame and DeLuca [133]. Overdosing on vitamin A, according to Lima and Souza [55], lowered egg production in layers.

## 10. Conclusions

From the published review of the literature, it is concluded that the supplementation of vitamin A in optimum concentrations supports growth, feed efficiency, egg production, and quality in poultry. Furthermore, vitamin A improved immunity, hatchability, and congenital malformations. However, the literature is not sufficiently mature to allow for definitive conclusions to be drawn. Opinions are divided on the effects of vitamin A or provitamin A supplementation on the production performance and health benefits in poultry. Probably, the beneficial effects of vitamin A and their precursors are highly dependent on the dose, duration, strains of birds, purity of the product, experimental design, and other environmental factors. Moreover, little information is available on the mechanism through which vitamin A and provitamin A produce these beneficial effects. Future studies should be focused on the molecular mechanism in various poultry species and are recommended to fully understand the health-promoting effects of vitamin A and provitamin A.

## Figures and Tables

**Table 1:** The NRC [24] requirements of vitamin A in poultry.

Sr. No.	Species	Requirement
1	Broiler	1500 IU per kg
2	Growing layers	1500 IU per kg
3	Growing Gees	1500 IU per kg
4	Turkeys	5000 IU per kg
5	Growing ducks	2500 IU per kg
6	Breeder ducks	4000 IU per kg
7	Growing Japanese quails	1650 IU per kg
8	Breeder quails	3300 IU per kg

**Table 2:** Dietary supplementation of vitamin A on production performance of poultry.

Parameter	Species	Dietary Supplementation	Effect	Reference
*Feed Intake*				
	Layers	9000 IU/kg	Increased	[31]
	Layer	5000 IU/kg (T5) to 25,000 IU/kg (T25)	No effect	[35]
	Layer	16,000 IU/kg	Increased	[37]
	Broiler	0 or 3000 IU/kg	Decreased	[38]
	Broiler	5000 IU/kg	Increased	[29]
	Layers	8 mg/kg canthaxanthin	Increased	[43]
*Body weight*				
	Broilers chicks	15,000 IU/kg	Increased	[44]
	Layer	16,000 IU/kg	Increased	[37]
	Growing Ducks	Above 2500 IU/kg	Decreased	[45]
	Growing Ducks	0 to 2500 IU/kg	Increased	[45]
	Broiler	2645 to 7935 IU/kg	Decreased	[46]
	Broiler	35,512 and 65,512 IU/kg	Decreased	[47,48]
	Broiler	0 or 3000 IU/kg	Decreased	[38]
*Carcass traits/Meat quality*				
Drip loss	Broiler	10,00,000 IU/kg	Decreased	[49]
Shear force	Goslings	21,600 IU/kg	Increased	[29]
pH	Breeder laying hens	9000 IU/kg	Increased	[50]
pH	Broilers chicks	40 mg/kg lycopene	Increased chick body weight	[51]
Redness	Broiler	15,000 IU/kg	Increased	[44]
Carcass traits	Broiler	3208~6208 IU/kg	Increased	[52]
Carcass quality	Broiler	10,000 IU/kg	Increased	[53]
Relative liver weight		15,000 IU per kg	Decreased	[54]
*Hatchability and reproduction*				
Hatching performance	Broiler	Overdose	Decreased	[55]
Hatchability	Broiler	10,000 to 15,000 IU/kg	Decreased	[56]
Reproductive performance	Layer	12,000 IU/kg	Increased	[38]
Hatchability and reproduction	Layers	16,000 IU/kg	Increased	[37]
Hatching capacity	Layers	9000 IU/kg	Increased	[31]
*Egg yield and quality*				
	Layers	9000 IU/kg	Increased	[31]
	Layer	5000 to 25,000 IU/kg	No effect	[35]
	Layer	210,000 and 410,000 IU/kg	Decreased	[57]
	Layer	16,000 IU/kg	Increased	[37]
Egg production	Layer	Overdose	Decreased	[55]
Egg production	Layers	24,000 IU/kg	No effect	[58]
Laying performance	Layers	9000 IU/kg	Increased	[31]
Egg yield	Broiler	10,000 to 15,000 IU/kg	Decreased	[56]
Egg yield	Layers	24,000 IU/kg	No effect	[58]
Efficiency of vitamin A transfer from diet to egg	Layer	8000 IU/kg	No effect	[59]
Egg production	Layer	210,000 and 410,000 IU/kg	Decreased	[57]
No. of eggs produced	Layer	16,000 IU/kg	Increased	[37]
Egg size	Broiler	10,000 to 15,000 IU/kg	Decreased	[56]
Egg production	Layer	5000 to 25,000 IU/kg	No effect	[35]
Egg yolk mass and egg weight	Layers	8 mg/kg canthaxanthin	Increased	[43]
Egg yolk CX concentration	Broiler breeders	6 mg/kg canthaxanthin	Increased	[60]

**Table 3:** Effect of vitamin A on health performance of poultry.

Parameter	Species	Dietary Supplementation	Effect	Reference
*Antioxidant activity*				
Serum MDA	Broiler	15,000 IU per kg	Decreased	[44]
Plasma GPx	Broiler	4500 IU per kg	Decreased	[70]
Serum activity of GPx activity	Broiler	Maternal VA 10,800 IU/kg or dietary VA 5000 IU/kg	Increased	[29]
Activities of SOD, GPx, CAT	Broiler	3000 IU/kg	Increased	[52]
Serum MDA	Broiler	3000 IU/kg	Decreased	[52]
Activity of GPx and CAT	Goslings	9000 IU/kg	Increased	[50]
MDA level	Goslings	9000 IU/kg	Decreased	[50]
Liver SOD, CAT, GPx	Japanese quails	25 or 50 g/kg tomato powder	Increase	[71]
Liver TOC	Duck	6 mg/kg canthaxanthin	Increase	[72]
MDA	Broilers	5% lycopene	Decrease	[73]
Oxidative stability	Broilers	75 mg/kg lycopene	Increase	[74]
*Disease/Disorder*				
TD and TD index	Broiler	65,512 IU/kg	Increased	[47,48]
Incidence of clinical rickets	Turkey	44,000 IU/kg	Increased	[75]
AA	Layer	75,000 IU/kg	Increased	[76,77]
Swelling of the inoculated left inter tarsal joints	Layer	75,000 IU/kg	Increased	[76]
AA and SAA	Layer	75,000 IU/kg	Increased	[76]
Conjunctivitis and impaired bone development	Growing Broiler	100,000 IU/kg	Decreased	[49]
*E. coli* caused mortality	Broiler chicks	60,000 IU/kg	Decreased	[78]
TD incidence and TD index	Broiler	65,512 IU/kg	Decreased	[47]
*Biochemical Activity*				
T_4_	Layer	8000 IU/kg	Decreased	[37]
ALT	Layer	16,000 IU/kg	Decreased	[37]
AST	Layer	16,000 IU/kg	Increased	[37]
HDL, LDL	Broiler	12,000 IU/kg	Increased	[38]
Thyroid and growth hormones	Goslings	Maternal 8000; 12,000 IU/kg	Increased	[50]
Insulin content of the offspring	Goslings	maternal VA 4000, 8000, 12,000, and 16,000 IU/kg	Increased	[50]
GPx, SOD, ALT, AST, ALP and lipase concentration	Broilers	5% lycopene	Increase	[69]
*Hematological Indices*				
Heterophils, lymphocytes, monocytes, eosinophil and basophils etc.	Layer	8000 and 16,000 IU/kg	No effect	[37]
Peripheral T lymphocyte proliferation	Broiler chicks	3000 to 12,000 IU/kg	Increased	[79,80,81]
Packed cell volume	Layer	8000 IU/kg	Decreased	[37]
*Egg/plasma retinol concentration*				
	Layer	15,000 IU, 20,000 and 25,000 IU/kg	Increased	[35]
	Goslings	9000 IU/kg	Increased	[50]
	Growing Ducks	2500 IU/kg or above	Increased	[40]
	Layer	75,000 IU/kg	Decreased	[76]
*Immune response*				
Antibody response to NDV	Layers	24,000 IU/kg	No effect	[58]
Antibody response to NDV	Layer	10,000 IU/kg	Increased	[39]
Antibody response to NDV	Broiler	5000 IU/kg	Increased	[29]
Antibody response to NDV	Broiler	1500 to 15,000 IU/kg	Increased	[54]
Antibody response to NDV	Broiler	15,000 IU/kg	Decreased	[55]
T lymphocyte and α-naphthyl acetate esterase (ANAE)-positive cells proportion	Layers	9000 IU/kg	Increased	[31]
Ratio of liver and bursa of Fabricius	Broiler	5000 IU/kg	Increased	[29]
Mucin and IgA levels in the bronchoalveolar lavage fluid (BALF)	Broiler chicks	1500, 6000 or 12,000 IU/kg	Increased	[82]
T-cell proliferation	Broiler chicks	6660 μg/kg	Increased	[81]
Th2 immune response	Broiler	5400 and 21,600 IU/kg	Increased	[54]
Natural killer activity and levels of percentage of cells expressing class II MHC antigens	Broiler	15,000 IU per kg	No effect	[54]
Lymphocyte responses to concanavalin A and pokeweed	Broiler	1500 to 15,000	Decreased	[54]
Lymphocyte responses to Mitogens	Broiler	15,000 IU per kg	Decreased	[54]
Plasma vitamin A levels	Layers	24,000 IU/kg	No effect	[58]
Average phytohemagglutinin (PHA) responses of birds	Broiler	1500 IU per kg	No effect	[54]
Heterophil/ lymphocyte ratio	Layer	75,000 IU/kg	Decreased	[76,83]
Expression of IL-1β	Broiler	5400 or 10,800 IU/kg	Increased	[29]
Expression of TNF-α	Broiler	10,800 IU/kg	Increased	[29]
Expression of IFN-g	Broiler	21,600 IU/kg	Decreased	[29]
Expression of IL-2	Broiler	5400 or 5000 IU/kg	Increased	[29]
Expression of IFN-γ	Broiler	Maternal vitamin A 21,600 IU/kg + dietary vitamin A 5000 IU/kg	Decreased	[29]
Liver Nrf2 levels	Japanese quails	25 or 50 g/kg tomato powder	Increase	[71]
Interdigital skin reactions to phytohemagglutinin (PHA) and CD4:CD8 T lymphocyte ratios	Broiler	400 IU/kg	Decreased	[54]
*Miscellaneous*				
Skin pigmentation	Broiler	5512 and 65,512 IU/kg	Decreased	[47]
	Broiler	35,512 IU/kg	No effect	[47]
Bone ash	Turkey			
Egg yolk tocopherol	Layer	44,000 IU/kg	Decreased	[75]
Enrichment of egg yolk with vitamin A	Layer	15,000, 20,000 and 25,000 IU/kg	Decreased	[35]

CX: Canthaxanthin; GPx: Glutathione peroxidase; SOD: Superoxide dismutase; CAT: Catalase; MDA: Malondialdehyde; TOC: Total oxidant capacity; TD: Dyschondroplasia; AA: amyloid arthropathy; SAA: amyloid-related precursor protein; T_4_: Thyroid hormone; ALT: Alanine transaminase; AST: Aspartate aminotransferase; HDL: high-density lipoprotein; LDL: low-density lipoprotein concentration; ALP: alkaline phosphatase.

## References

[1] Sommer A. (2008). Vitamin a deficiency and clinical disease: An historical overview. J. Nutr..

[2] Ross S.A., Mccaffery P.J., Drager U.C., De Luca L.M. (2000). Retinoids in embryonal development. Physiol. Rev..

[3] Stepp W. (1909). Experiments on feeding with lipoid-free food. Biochem. Z..

[4] McCollum E.V., Davies M. (1913). The necessity of certain lipids during growth. J. Biol. Chem..

[5] Drummond J.C., Coward K.H. (1920). Researches on the Fat-soluble Accessory Factor (Vitamin A). VI: Effect of Heat and Oxygen on the Nutritive Value of Butter. Biochem. J..

[6] Von Lintig J., Vogt K. (2000). Filling the gap in vitamin A research: Molecular identification of an enzyme cleaving β-carotene to retinal. J. Biol. Chem..

[7] Send R., Sundholm D. (2007). Stairway to the conical intersection: A computational study of the retinal isomerization. J. Phys. Chem. A.

[8] Nabi F., Arain M.A., Rajput N., Alagawany M., Soomro J., Umer M., Liu J. (2020). Health benefits of carotenoids and potential application in poultry industry: A review. J. Anim. Physiol. Anim. Nutr..

[9] Palace V.P., Khaper N., Qin Q., Singal P.K. (1999). Antioxidant potentials of vitamin A and carotenoids and their relevance to heart disease. Free Radic. Biol. Med..

[10] Sporn M.B., Dunlop N.M., Newton D.L., Smith J.M. (1976). Prevention of chemical carcinogenesis by vitamin A and its synthetic analogs (retinoids). Fed. Proc..

[11] Gerster H. (1997). Vitamin A—Functions, dietary requirements and safety in humans. Int. J. Vitam. Nutr. Res..

[12] Chambon P. (1996). A decade of molecular biology of retinoic acid receptors. FASEB J..

[13] Sahin N., Sahin K., Kucuk O. (2001). Effects of vitamin E and vitamin A supplementation on performance, thyroid status, and serum concentrations of some metabolites and minerals in broilers reared under heat stress (32 °C). Vet. Med..

[14] Castaneda M.P., Hirschler E.M., Sams A.R. (2005). Skin pigmentation evaluation in broilers fed natural and synthetic pigments. Poult. Sci..

[15] Liu G.D., Hou G.Y., Wang D.J., Lv S.J., Zhang X.Y., Sun W.P., Yang Y. (2008). Skin pigmentation evaluation in broilers fed different levels of natural okra and synthetic pigments. J. Appl. Poult. Res..

[16] Karadas F., Erdoğan S., Kor D., Oto G., Uluman M. (2016). The effects of different types of antioxidants (Se, vitamin E and carotenoids) in broiler diets on the growth performance, skin pigmentation and liver and plasma antioxidant concentrations. Braz. J. Poult. Sci..

[17] Johnson B.M., Charman W.N., Porter C.J. (2002). An in vitro examination of the impact of polyethylene glycol 400, pluronic P85, and vitamin E da-tocopheryl polyethylene glycol 1000 succinate on P-glycoprotein efflux and enterocyte-based metabolism in excised rat intestine. AAPS Pharmsci.

[18] Krinsky N.I., Johnson E.J. (2005). Carotenoid actions and their relation to health and disease. Mol. Asp. Med..

[19] Del Campo J.A., García-González M., Guerrero M.G. (2007). Outdoor cultivation of microalgae for carotenoid production: Current state and perspectives. Appl. Microbiol. Biotechnol..

[20] Jackson H., Braun C.L., Ernst H. (2008). The chemistry of novel xanthophyll carotenoids. Am. J. Cardiol..

[21] Rajput I.R., Li L.Y., Xin X., Wu B.B., Juan Z.L., Cui Z.W., Li W.F. (2013). Effect of *Saccharomyces boulardii* and *Bacillus subtilis* B10 on intestinal ultrastructure modulation and mucosal immunity development mechanism in broiler chickens. Poult. Sci..

[22] Harrison E.H. (2005). Mechanisms of digestion and absorption of dietary vitamin A. Annu. Rev. Nutr..

[23] McDowell L.R., Ward N.E. (2008). Optimum vitamin nutrition for poultry. Int. Poult. Prod..

[24] NRC, National Research Council (1994). Nutrient Requirements of Poultry.

[25] Olson J.A. (1984). Serum levels of vitamin A and carotenoids as reflectors of nutritional status. J. Natl. Cancer Inst..

[26] Tanumihardjo S.A., Howe J.A. (2005). Twice the amount of α-carotene isolated from carrots is as effective as β-carotene in maintaining the vitamin A status of Mongolian gerbils. J. Nutr..

[27] Scott M.I., Neshem M.C., Young R.J. (1982). Nutrition of Chicken.

[28] Ahmad S.M., Haskell M.J., Raqib R., Stephensen C.B. (2009). Markers of innate immune function are associated with vitamin a stores in men. J. Nutr..

[29] Wang Y., Li L., Gou Z., Chen F., Fan Q., Lin X., Jiang S. (2020). Effects of maternal and dietary vitamin A on growth performance, meat quality, antioxidant status, and immune function of offspring broilers. Poultr. Sci..

[30] Friedman A., Sklan D. (1997). Effects of retinoids on immune responses in birds. World’s Poult. Sci. J..

[31] Lin H., Wang L.F., Song J.L., Xie Y.M., Yang Q.M. (2002). Effect of dietary supplemental levels of vitamin A on the egg production and immune responses of heat-stressed laying hens. Poult. Sci..

[32] Khoramabadi V., Akbari M.R., Khajali F., Noorani H., Rahmatnejad E. (2014). Influence of xylanase and vitamin A in wheat-based diet on performance, nutrients digestibility, small intestinal morphology and digesta viscosity in broiler chickens. Acta Sci..

[33] Stanquevis C.E., de Paula V.R.C., Zancanela V.T., Benites M.I., Finco E.M., de Aquino D.R., Rodrigues T.P.P.M., Marcato S.M. (2022). Levels of vitamin A supplementation for growing meat-type quails. Emerg. Anim. Species.

[34] Chen F., Jiang Z., Jiang S., Li L., Lin X., Gou Z., Fan Q. (2016). Dietary vitamin A supplementation improved reproductive performance by regulating ovarian expression of hormone receptors, caspase-3 and Fas in broiler breeders. Poult. Sci..

[35] Mendonca C.X., Almeida C.R.M., Mori A.V., Watanabe C. (2002). Effect of dietary vitamin A on egg yolk retinol and tocopherol levels. J. Appl. Poult. Res..

[36] Safarizadeh A., Zakeri A. (2013). The effect of vitamin A and complex of vitamin E and selenium on growth factors and Humoral immunity in broiler chickens. Eur. J. Exp. Biol..

[37] Abd El-Hack M.E., Mahrose K., Askar A.A., Alagawany M., Arif M., Saeed M., Abbasi F., Soomro R.N., Siyal F.A., Chaudhry M.T. (2017). Single and Combined Impacts of Vitamin A and Selenium in Diet on Productive Performance, Egg Quality, and Some Blood Parameters of Laying Hens During Hot Season. Biol. Trace Elem. Res..

[38] Savaris V.D., Broch J., de Souza C., Junior N.R., de Avila A.S., Polese C., Kaufmann C., de Oliveira Carvalho P.L., Pozza P.C., Vieites F.M. (2021). Effects of vitamin A on carcass and meat quality of broilers. Poult. Sci..

[39] Kaya Ş., Umucalilar H., Haliloĝlu S., İpek H. (2001). Effect of dietary vitamin A and zinc on egg yield and some blood parameters of laying hens. Turk. J. Vet. Anim. Sci..

[40] Feng D., Wang X., Li E., Bu X., Qiao F., Qin J., Chen L. (2019). Dietary Aroclor 1254-induced toxicity on antioxidant capacity, immunity and energy metabolism in Chinese mitten crab Eriocheir sinensis: Amelioration by vitamin A. Front. Physiol..

[41] Tian Y., Nichols R.G., Cai J., Patterson A.D., Cantorna M.T. (2018). Vitamin A deficiency in mice alters host and gut microbial metabolism leading to altered energy homeostasis. J. Nutr. Biochem..

[42] Farhat K., Bodart G., Charlet-Renard C., Desmet C.J., Moutschen M., Beguin Y., Baron F., Melin P., Quatresooz P., Parent A.S. (2018). Growth hormone (GH) deficient mice with GHRH gene ablation are severely deficient in vaccine and immune responses against *Streptococcus pneumoniae*. Front. Immunol..

[43] Umar Faruk M., Roos F.F., Cisneros-Gonzalez F. (2018). A meta-analysis on the effect of canthaxanthin on egg production in brown egg layers. Poultr. Sci..

[44] Kucuk O., Sahin N., Sahin K. (2003). Supplemental zinc and Vitamin A can alleviate negative effects of heat stress in broiler chickens. Biol. Trace Elem. Res..

[45] Feng Y.L., Xie M., Tang J., Huang W., Zhang Q., Hou S.S. (2019). Effects of vitamin A on growth performance and tissue retinol of starter White Pekin ducks. Poult. Sci..

[46] Panda B., Combs G.F., DeVolt H.M. (1964). Studies on coccidiosis and vitamin A nutrition of broilers. Poult. Sci..

[47] Li J., Bi D., Pan S., Zhang Y., Zhou D. (2008). Effects of high dietary vitamin A supplementation on tibial dyschondroplasia, skin pigmentation and growth performance in avian broilers. Res. Vet. Sci..

[48] Jensen L.S., Fletcher D.L., Lilburn M.S., Akiba Y. (1983). Growth depression in broiler chicks fed high Vitamin A levels. Nutr. Rep. Int..

[49] Wolbach S.B., Hegsted D.M. (1952). Hypervitaminosis A and the skeleton of growing chicks. Arch. Pathol..

[50] Liang J.R., Xiao X., Yang H.M., Wang Z.Y. (2021). Assessment of vitamin A requirement of gosling in 0–28 d based on growth performance and bone indexes. Poult. Sci..

[51] Sun B., Chen C., Wang W., Ma J., Xie Q., Gao Y., Chen F., Zhang X., Bi Y. (2015). Effects of lycopene supplementation in both maternal and offspring diets on growth performance, antioxidant capacity and biochemical parameters in chicks. J. Anim. Physiol. Anim. Nutr..

[52] Hong P., Jiang Z.Y., Jiang S.Q., Zhou G.L., Zheng C.T., Lin Y.C. (2013). VA supplemental level: Effects on growth performance and antioxidant Parameters of yellow-feathered broilers aged from 43 to 63 Days. Chin. J. Anim. Nutr..

[53] Li X.H. (2006). Effect of Vitamin A, E and C on the Production Performance, Meat Quality and Serum Biochemical Parameter in Broiler chicks. Master’s Thesis.

[54] Lessard M., Hutchings D., Cave N.A. (1997). Cell-mediated humoral immune responses in broiler chickens maintained on diets containing different levels of vitamin A. Poult. Sci..

[55] Lima H.J.D., Souza L.A.Z. (2018). Vitamin A in the diet of laying hens: Enrichment of table eggs to prevent nutritional deficiencies in humans. World’s Poult. Sci. J..

[56] Yuan J., Roshdy A.R., Guo Y., Wang Y., Guo S. (2014). Effect of dietary vitamin A on reproductive performance and immune response of broiler breeders. PLoS ONE.

[57] March B.E., Coates V., Goudie C. (1972). Delayed hatching time of chicks from dams fed excess vitamin A and from eggs injected with vitamin A. Poult. Sci..

[58] Coşkun B., Inal F., Celik I., Erganiş O., Tiftik A.M., Kurtoglu F., Kuyucuoglu Y., Ok U. (1998). Effects of dietary levels of vitamin A on the egg yield and immune responses of laying hens. Poult. Sci..

[59] Squires M.W., Naber E.C. (1993). Vitamin profiles of eggs as indicators of nutritional status in the laying hen: Vitamin A study. Poult. Sci..

[60] Rosa A.P., Scher A., Sorbara J.O., Boemo L.S., Forgiarini J., Londero A. (2012). Effects of canthaxanthin on the productive and reproductive performance of broiler breeders. Poult. Sci..

[61] Kenny M., Kemp C. (2005). Breeder nutrition and chick quality. Int. Hatchery Pract..

[62] Yang H., Liang J., Dai H., Wan X., Wang Z. (2020). Effects of vitamin A supplementation in the diet of breeding geese on offspring intestinal tissue morphology and immune performance. Asian-Austral. J. Anim. Sci..

[63] Aburto A., Britton W.M. (1998). Effects of different levels of vitamins A and E on the utilization of cholecalciferol by broiler chickens. Poult. Sci..

[64] Langi P., Kiokias S., Varzakas T., Proestos C. (2018). Carotenoids: From plants to food and feed industries. Microb. Carotenoids.

[65] Zhu C., Farré G., Zanga D., Lloveras J., Michelena A., Ferrio J.P., Voltas J., Slafer G., Savin R., Albajes R. (2018). High-carotenoid maize: Development of plant biotechnology prototypes for human and animal health and nutrition. Phytochem. Rev..

[66] Yatao X., Saeed M., Kamboh A., Arain M., Ahmad F., Suheryani I., Shah Q.A., Chao S. (2018). The potentially beneficial effects of supplementation with hesperidin in poultry diets. World’s Poultr. Sci. J..

[67] Xiao X., Liang J.R., Yang H.M., Wan X.L., Wang Z.Y. (2021). Vitamin A deficiency or critical excess has negative effects on the growth performance, slaughter performance, and meat quality of goslings. Anim. Feed. Sci. Technol..

[68] Combs G.F., McClung J.P. (2016). The Vitamins: Fundamental Aspects in Nutrition and Health.

[69] Leeson S. (2007). Vitamin requirements: Is there basis for re-evaluating dietary specifications?. World Poult. Sci. J..

[70] Mahmoud K.Z., Hijazi A.A. (2007). Effect of vitamin A and/or E on plasma enzymatic antioxidant systems and total antioxidant capacity of broiler chickens challenged with carbon tetrachloride. J. Anim. Physiol. Anim. Nutr..

[71] Sahin K., Orhan C., Akdemir F., Tuzcu M., Ali S., Sahin N. (2011). Tomato powder supplementation activates Nrf-2 via ERK/Akt signaling pathway and attenuates heat stress-related responses in quails. Anim. Feed. Sci. Technol..

[72] Ren Z.Z., Zeng Q.F., Wang J.P., Ding X.M., Bai S.P., Su Z.W., Zhang K.Y. (2018). Effects of maternal dietary canthaxanthin and 25-hydroxycholecalciferol supplementation on antioxidant status and calcium-phosphate metabolism of progeny ducks. Poult. Sci..

[73] Hosseini-Vashan S.J., Golian A., Yaghobfar A. (2016). Growth, immune, antioxidant, and bone responses of heat stress-exposed broilers fed diets supplemented with tomato pomace. Int. J. Biometeorol..

[74] Englmaierová M., Bubancová I., Vít T., Skrivan M. (2011). The effect of lycopene and vitamin E on growth performance, quality and oxidative stability of chicken leg meat. Czech J. Anim. Sci..

[75] Stevens V.I., Blair R., Riddell C. (1983). Dietary levels of fat, calcium and vitamins A and D3 as contributory factors to rickets in poults. Poult. Sci..

[76] Sevimli A., Misirlioglu D., Polat Ü., Yalçin M., Akkoç A., Uguz C. (2005). The effects of vitamin A, pentoxyfylline and methylprednisolone on experimentally induced amyloid arthropathy in brown layer chicks. Avian Pathol..

[77] Sevimli A., Mısırlıoglu D., Özakin C. (2004). The enhancing effect of Vitamin A on the occurrence of amyloid arthropathy in laying chickens infected with *Enterococcus faecalis*. Turk. J. Vet. Anim. Sci..

[78] Tengerdy R.P., Brown J.C. (1977). Effect of vitamin E and A on humoral immunity and phagocytosis in E. coli infected chicken. Poultr. Sci..

[79] Friedman A., Sklan D. (1989). Impaired T lymphocyte immune response in vitamin A depleted rats and chicks. Br. J. Nutr..

[80] Friedman A., Meidovsky A., Leitner G., Sklan D. (1991). Decreased resistance and immune response to *Escherichia coli* infection in chicks with low or high intakes of vitamin A. J. Nutr..

[81] Sklan D., Melamed D., Friedman A. (1994). The effect of varying levels of dietary vitamin A on immune response in the chick. Poult. Sci..

[82] Fan X., Liu S., Liu G., Zhao J., Jiao H., Wang X., Lin H. (2015). Vitamin A deficiency impairs mucin expression and suppresses the mucosal immune function of the respiratory tract in chicks. PLoS ONE.

[83] Zekerias B., Landman W.J.M., Tooten P.C.T., Gruys E. (2000). Leucocyte responses in two breeds of layer chicken that differ in susceptibility to induced amyloid arthropathy. Vet. Immunol. Immunopathol..

[84] Fu Z., Kato H., Sugahara K., Kubo T. (2000). Retinoic acid accelerates the development of reproductive organs and egg position in Japanese quail (*Coturnix coturnix japonica*). Biol. Reprod..

[85] Ninomiya Y., Arao Y., Kometani T., Hiwatashi S., Yamasaki T., Erikawa T., Yamaguchi H., Hasegawa T., Masushige S., Kato S. (1996). Vitamin A is involved in estrogen induced cell proliferation but not in cytodifferentiation of the chicken oviduct. J. Endocrinol..

[86] Onagbesan O.M., Decuypere E., Leenstra F., Ehlhardt D.A. (1999). Differential effects of amount of feeding on cell proliferation and progesterone production in response to gonadotrophins and insulin-like growth factor I by ovarian granulosa cells of broiler breeder chickens selected for fatness or leanness. J. Reprod. Fertil..

[87] Onagbesan O.M., Vleugels B., Buys N., Bruggeman V., Safi M., Decuypere E. (1999). Insulin-like growth factors in the regulation of avian ovarian functions. Domest. Anim. Endocrinol..

[88] De Carvalho P.R., Cipolli K.M.V.A.B., Ormenese R.D.C.S.C., Carvalho P.R.N., da Silva M.G. (2009). Supplementation carotenoid compounds derived from seed integral ground annatto (*Bixa orellana* L.) in the feed laying hens to produce eggs special. Pak. J. Nutr..

[89] Hamelin C., Martinez-Aleson R., Martínez F. Influence of feed carotenoids on carcass and shank pigmentation of yellow chickens. Proceedings of the XXI European Symposium on the Quality of Poultry Meat and the XV European Symposium on the Quality of Eggs and Egg Products.

[90] Liu Y.Q., Davis C.R., Schmaelzle S.T., Rocheford T., Cook M.E., Tanumihardjo S.A. (2012). beta-Cryptoxanthin biofortified maize (*Zea mays*) increases beta-cryptoxanthin concentration and enhances the color of chicken egg yolk. Poult. Sci..

[91] Titcomb T., Kaeppler M., Cook M., Simon P., Tanumihardjo S. (2019). Carrot leaves improve color and xanthophyll content of egg yolk in laying hens but are not as effective as commercially available marigold fortificant. Poult. Sci..

[92] Czauderna M., Białek M., Białek A., Śliwiński B., Brzóska F. (2019). Chemical form of dietary selenium affects the fatty acids profile and oxidative stability of muscles of broilers supplemented with lycopene and oils. Eur. J. Lipid Sci. Technol..

[93] Panaite T.D., Nour V., Vlaicu P.A., Ropota M., Corbu A.R., Saracila M. (2019). Flaxseed and dried tomato waste used together in laying hens diet. Arch. Anim. Nutr..

[94] Krinsky N.I. (2001). Carotenoids as antioxidants. Nutrition.

[95] Zhang L., Hou Y., Ma Z., Xie J., Fan J., Jiao Y., Wang F., Han Z., Liu S., Ma D. (2023). Effect of oral vitamin A supplementation on host immune response to infectious bronchitis virus infection in SPF chicken. Poult. Sci..

[96] Guo S., He L., Zhang Y., Niu J., Li C., Zhang Z., Li P., Ding B. (2023). Effects of Vitamin A on Immune Responses and Vitamin A Metabolism in Broiler Chickens Challenged with Necrotic Enteritis. Life.

[97] Catharine R.A. (2012). Vitamin A and retinoic acid in T cell–related immunity. Am. J. Clin. Nutr..

[98] Sijtsma S.R., West C.E., Rombout J.H.W.M., Van der Zijpp A.J. (1989). The interaction between vitamin A status and Newcastle disease virus infection in chickens. J. Nutr..

[99] Davis C.Y., Sell J.L. (1989). Immunoglobulin concentrations in serum and tissues of vitamin A-deficient broiler chicks after Newcastle disease virus vaccination. Poult. Sci..

[100] Ferket P.R., Qureshi M.A. (1992). Performance and immunity of heat-stressed broilers fed vitamin- and electrolyte supplemented drinking water. Poult. Sci..

[101] Saino N., Stradi R., Ninni P., Pini E., Moller A.P. (1999). Carotenoid plasma concentration, immune profile, and plumage ornamentation of male barn swallows (*Hirundo rustica*). Am. Nat..

[102] Saeed M., Yatao X., Hassan F.-U., Arain M.A., Abd El-Hack M.E., Noreldin A.E., Sun C. (2018). Influence of graded levels of l-theanine dietary supplementation on growth performance, carcass traits, meat quality, organs histomorphometry, blood chemistry and immune response of broiler chickens. Int. J. Mol. Sci..

[103] Nogareda C., Moreno J.A., Angulo E., Sandmann G., Portero M., Capell T., Zhu C., Christou P. (2016). Carotenoid-enriched transgenic corn delivers bioavailable carotenoids to poultry and protects them against coccidiosis. Plant Biotechnol. J..

[104] Koutsos E.A., Clifford A.J., Calvert C.C., Klasing K.C. (2003). Maternal carotenoid status modifies the incorporation of dietary carotenoids into immune tissues of growing chickens (*Gallus gallus domesticus*). J. Nutr..

[105] Olson J., Ward N., Koutsos E. (2008). Lycopene incorporation into egg yolk and effects on laying hen immune function. Poult. Sci..

[106] Bennedsen M., Wang X., Willén R., Wadström T., Andersen L.P. (1999). Treatment of *H. pylori* infected mice with antioxidant astaxanthin reduces gastric inflammation, bacterial load and modulates cytokine release by splenocytes. Immunol. Lett..

[107] Guerin M., Huntley M.E., Olaizola M. (2003). *Haematococcus astaxanthin*: Applications for human health and nutrition. Trends Biotechnol..

[108] Arain M.A., Mei Z., Hassan F., Saeed M., Alagawany M., Shar A., Rajput I. (2018). Lycopene: A natural antioxidant for prevention of heat-induced oxidative stress in poultry. World’s Poult. Sci. J..

[109] Palozza P., Catalano A., Simone R.E., Mele M.C., Cittadini A. (2012). Effect of lycopene and tomato products on cholesterol metabolism. Ann. Nutr. Metab..

[110] Kaulmann A., Bohn T. (2014). Carotenoids, inflammation, and oxidative stress—Implications of cellular signaling pathways and relation to chronic disease prevention. Nutr. Res..

[111] Aydelotte M.B. (1963). The effects of vitamin A and citral on epithelial differentiation in vitro. 1. The chick tracheal epithelium. J. Embryol. Exp. Morphol..

[112] Khan R.U., Rahman Z.U., Javed I., Muhammad F. (2013). Supplementation of vitamins, probiotics and proteins on oxidative stress, enzymes and hormones in post-moulted male broiler breeder. Arch. Tierz..

[113] Surai P.F. (2002). Selenium in poultry nutrition 2. Reproduction egg and meat quality and practical applications. World’s Poultr. Sci. J..

[114] McDowell L.R., Wilkinson N., Madison R., Felix T. Vitamins and minerals functioning as antioxidants with supplementation considerations. Proceedings of the Florida Ruminant Nutrition Symposium.

[115] Lee C.-Y., Lee B.-D., Na J.-C., An G. (2010). Carotenoids accumulation and their antioxidant activity in spent laying hens as affected by polarity and feeding period. Asian-Austral. J. Anim. Sci..

[116] Hammershøj M., Kidmose U., Steenfeldt S. (2010). Deposition of carotenoids in egg yolk by short-term supplement of coloured carrot (*Daucus carota*) varieties as forage material for egg-laying hens. J. Sci. Food Agric..

[117] Bédécarrats G.Y., Leeson S. (2006). Dietary lutein influences immune response in laying hens. J. Appl. Poult. Res..

[118] Liang J.R., Dai H., Yang H.M., Yang Z., Wang Z.Y. (2019). The effect of dietary vitamin A supplementation in maternal and its offspring on the early growth performance, liver vitamin A content, and antioxidant index of goslings. Poult. Sci..

[119] Rao A.V., Rao L.G. (2007). Carotenoids and human health. Pharmacol. Res..

[120] Changxing L., Chenling M., Alagawany M., Jianhua L., Dongfang D., Gaichao W., Wenyin Z., Syed S.F., Arain M.A., Saeed M. (2018). Health benefits and potential applications of anthocyanins in poultry feed industry. World’s Poult. Sci. J..

[121] Yuan J.P., Peng J., Yin K., Wang J.H. (2011). Potential health-promoting effects of astaxanthin: A high-value carotenoid mostly from microalgae. Mol. Nutr. Food Res..

[122] Giovannucci E. (2002). A review of epidemiologic studies of tomatoes, lycopene, and prostate cancer. Exp. Biol. Med..

[123] Goralczyk R., Siler U. (2003). The role of lycopene in health and disease. Phytochem. Health Dis..

[124] Tufarelli V., Baghban-Kanani P., Azimi-Youvalari S., Hosseintabar-Ghasemabad B., Slozhenkina M., Gorlov I., Seidavi A., Ayaṣan D.K., Laudadio V. (2021). Effects of Horsetail (Equisetum arvense) and Spirulina (Spirulina platensis) Dietary Supplementation on Laying Hens Productivity and Oxidative Status. Animals.

[125] Karadas F., Grammenidis E., Surai P.F., Acamovic T., Sparks N.H. (2006). Effects of carotenoids from lucerne, marigold and tomato on egg yolk pigmentation and carotenoid composition. Br. Poult. Sci..

[126] Akdemir F., Orhan C., Sahin N., Sahin K., Hayirli A. (2012). Tomato powder in laying hen diets: Effects on concentrations of yolk carotenoids and lipid peroxidation. Br. Poult. Sci..

[127] Monaghan B.R., Schmitt F.O. (1932). The effects of carotene and of vitamin A on the oxidation of linoleic acid. J. Biol. Chem..

[128] Burton G.W., Ingold K.U. (1984). β-carotene: An unusual type of lipid antioxidant. Science.

[129] Stahl W., Sies H. (1996). Lycopene: A biologically important carotenoid for humans?. Arch. Biochem. Biophys..

[130] Nagy N.E., Holven K.B., Roos N., Senoo H., Kojima N., Norum K.R., Blomho V.R. (1997). Storage of vitamin A in extrahepatic stellate cells in normal rats. J. Lipid Res..

[131] Tesoriere L., Ciaccio M., Bongiorno A., Riccio A., Pintaudi A.M., Livrea M.A. (1993). Antioxidant activity of all-trans-retinol in homogeneous solution and in phophastidylcholine liposomes. Arch. Biochem. Biophys..

[132] Das N.P. (1989). Effects of vitamin A and its analogs on nonenzymatic lipid peroxidation in rat brain mitochondria. J. Neurochem..

[133] Clagett-Dame M., DeLuca H.F. (2002). The role of vitamin A in mammalian reproduction and embryonic development. Annu. Rev. Nutr..

